# Facet-Dependent Cuprous Oxide Nanocrystals Decorated with Graphene as Durable Photocatalysts under Visible Light

**DOI:** 10.3390/nano8060423

**Published:** 2018-06-11

**Authors:** Shou-Heng Liu, Jun-Sheng Lu

**Affiliations:** Department of Environmental Engineering, National Cheng Kung University, Tainan 70101, Taiwan; dalelu4404@gmail.com

**Keywords:** Cu_2_O, crystal facets, graphene sheets, photocatalytic degradation, durability

## Abstract

Three morphologies (octahedral, hierarchical and rhombic dodecahedral) of crystal Cu_2_O with different facets ({111}, {111}/{110}, and {110}) incorporating graphene sheets (denoted as o-Cu_2_O-G, h-Cu_2_O-G and r-Cu_2_O-G, respectively) have been fabricated by using simple solution-phase techniques. Among these photocatalysts, the r-Cu_2_O-G possesses the best photocatalytic performance of 98% removal efficiency of methyl orange (MO) with outstanding kinetics for 120 min of visible light irradiation. This enhancement is mainly due to the dangling “Cu” atoms in the highly active {110} facets, resulting in the increased adsorption of negatively charged MO. More importantly, the unique interfacial structures of Cu_2_O rhombic dodecahedra connected to graphene nanosheets can not only decrease the recombination of electron-hole pairs but also stabilize the crystal structure of Cu_2_O, as verified by a series of spectroscopic analyses (e.g., X-ray diffraction (XRD), X-ray photoelectron spectroscopy (XPS), scanning electron microscopy (SEM) and transmission electron microscopy (TEM)). The effective photocatalysts developed in this work could be applied to the efficient decolorization of negatively charged organic dyes by employing solar energy.

## 1. Introduction 

Metal oxide semiconductors have been intensively investigated for photocatalytic degradation of organic pollutants for many years [1,2,3]. However, these photocatalysts, e.g., TiO_2_ and ZnO (band gap ≅ 3.2 eV), can exhibit superior photocatalysis properties in the ultraviolet (UV) light region, leading to their limited practical application in wastewater treatments due to the restricted use of solar energy [4,5,6,7,8]. Cuprous oxide (Cu_2_O), which is a p-type semiconductor with a direct band gap of ca. 2.17 eV, has been widely studied as an efficient photocatalyst [9,10,11,12,13] because of its abundance, low cost, environmental-friendliness and good visible-light response. Nonetheless, the photocatalytic activity of Cu_2_O is constrained by fast recombination of the electron/hole (e/h) pairs [14] and has low durability [15]. Therefore, many studies have been dedicated to enhancing visible light-active photocatalysis by enhancing the segregation of electron-hole pairs. For instance, incorporating ions into the semiconductor [16,17,18,19,20,21], sensitization with dyer and surface complex [22,23,24], and coupling two or more semiconductors [25,26]. Moreover, well-defined facets of Cu_2_O which exhibit unique crystallographic properties related to different atomic terminated arrangements have been demonstrated to make a great improvement to photocatalysis [27,28,29]. The {110} facets of Cu_2_O were found to have a superior photocatalytic activity toward the degradation of methyl orange [27]. In addition, the Cu_2_O octahedra crystals consisting of {111} facets showed higher photocatalytic performance as compared to truncated cubic crystals abundant in {100} facets which has been attributed to the lower surface energy density of {100} facets than that of {111} facets [30]. In comparison to the pure Cu_2_O octahedra with {111} surfaces and pure cubes with {100} surfaces, the combination of 26-facet and 18-facet polyhedra with main {110} was observed to have better adsorption and photocatalytic activities [31]. However, the aforementioned Cu_2_O crystals may suffer from the deterioration of their crystal structure during long-term operation [32].

To further increase the stability of Cu_2_O nanocrystals, one of the possible methods was carried out by the incorporation of carbonaceous materials onto Cu_2_O [33,34,35]. Graphene is a well-known two-dimensional (2D) carbon material [36], which has distinguishing physiochemical properties such as a theoretical surface area (~2965 m^2^ g^−1^) [37], high intrinsic electron mobility (2 × 10^5^ cm^2^ V^−1^ s^−1^) [38], and exceptional chemical durability [39,40,41]. Owing to these unique properties, visible light-driven photocatalysts based on the synthesis of Cu_2_O-graphene composites [35,42,43] for fuel production and pollutant degradation have been proposed. However, limited studies have been reported in terms of different facets of Cu_2_O-graphene nanoheterostructures on their visible light-responsive activity and corresponding durability.

In this research, three different morphologies of Cu_2_O crystals with low-index facets ({111}, {111}/{110}, and {110}) decorated with graphene sheets were prepared via simple wet-chemical methods. These as-synthesized Cu_2_O crystals were thoroughly characterized by a variety of analytical spectroscopies and used as visible light-driven photocatalysts in the degradation of methyl orange (MO).

## 2. Experiment 

### 2.1. Photocatalyts Preparation

Typically, the synthesis of graphene oxide (GO) was carried out by referring to a modified route described earlier [44]. For preparation of octahedral Cu_2_O [27] and octahedral Cu_2_O-graphene, ca. 88 mL of deionized water or 1% GO solution was mixed with ca. 1 mL of copper(II) chloride solution and 0.87 g of sodium dodecyl sulfate (SDS) solids under vigorous stirring until the dissolution of the SDS powder. Then, 8.5 mL of 0.2 M NH_2_OH·HCl and 2.5 mL of 1.0 M NaOH solution were consecutively added to the resulting mixture. Lastly, the precipitate was treated by centrifugation and dried under a vacuum. The aforementioned photocatalysts prepared by using deionized water and 1% GO solution in the synthesis process were labelled as o-Cu_2_O and o-Cu_2_O-G, respectively.

In terms of the synthesis of hierarchical facets of Cu_2_O [30] and hierarchical facets of Cu_2_O-graphene, 48 mL of 1% GO solution was mixed well with 1 mL of copper(II) chloride solution. Afterward, 40 mL of ethylene glycol (EG) was introduced to the aforementioned mixture. Then, 24 mL of 0.1 M NH_2_OH·HCl was added to the above solution for 10 min. The resultant solution was moved to a Teflon-lined stainless steel autoclave, followed by ramping from room temperature to 180 °C for 1 h. The solid products were filtrated, washed and dried at ambient temperature. The aforementioned samples prepared without and with 1% GO solution in the synthesis process were denoted as h-Cu_2_O and h-Cu_2_O-G, respectively.

For the preparation of rhombic dodecahedral Cu_2_O [27] and rhombic dodecahedral Cu_2_O-graphene, ca. 34.6 mL of deionized water or 1% GO solution was heated to 34 °C in a water bath. Then, ca. 2.5 mL of copper(II) chloride solution (0.1 M) and 0.44 g of SDS solids were added into the above mixture with continuous stirring. While the SDS was dissolved, 2.5 mL of NaOH solution was introduced, followed by adding 12 mL of 0.1 M NH_2_OH·HCl to the resulting mixture. Finally, the precipitated products were separated by centrifuge, washed by using a water–ethanol mixture and dried. The aforementioned samples prepared by using deionized water and 1% GO solution in the synthesis process were denoted as r-Cu_2_O and r-Cu_2_O-G, respectively.

### 2.2. Characterizations of Photocatalyts

Powder X-ray diffraction (XRD) patterns of the samples were examined by using a PANalytical X’Pert PRO diffractometer with Cu Kα radiation (*λ* = 1.541 Å). The elemental compositions of samples were analysed by X-ray photoelectron spectroscopy (XPS) using a Kratos AXIS Ultra DLD spectrometer (Kratos Analytical Ltd., Stretford, Manchester, UK) and monochromated Al Kα X-ray source. The morphologies of the photocatlysts were observed through a scanning electron microscope (SEM, JEOL-7000F, JEOL Ltd., Akishima, Tokyo, Japan) with an accelerating voltage of 20 kV. In addition, to further study the detailed surface characteristics of the Cu_2_O nanocrystals, a high-resolution transmission electron microscopy (TEM, JEOL 2100F, JEOL Ltd., Akishima, Tokyo, Japan) was carried out. The UV-visible (UV-Vis) diffuse reflection spectra of the samples were collected on a UV-Vis spectrophotometer (Varian, Cary 100, Palo Alto, CA, USA).

### 2.3. Photocatalytic Degradation of Organic Pollutants

Photocatalytic degradation tests of MO were performed at 25 °C in a photoreactor (100 mL) described previously [35]. The test solution was prepared via mixing 5.0 mg of photocatalysts into 80 mL of MO solution (15 mg L^−1^). Prior to irradiation, the aqueous solution was kept stirring in the dark for 2 h to establish the adsorption equilibrium. The pH value of the solution was 5.6. Then, the photocatalytic reaction was performed by using a 300 W Xe lamp combined with a 420 nm cutoff filter as the light source. About 2 mL of aliquots was periodically withdrawn for every 30 min, centrifuged to separate solid samples, and the variations of concentration were measured by using a Hitachi UV-Visible (UV-Vis) spectroscope (Model U-2910).

## 3. Results and Discussion

**Figure 1:**
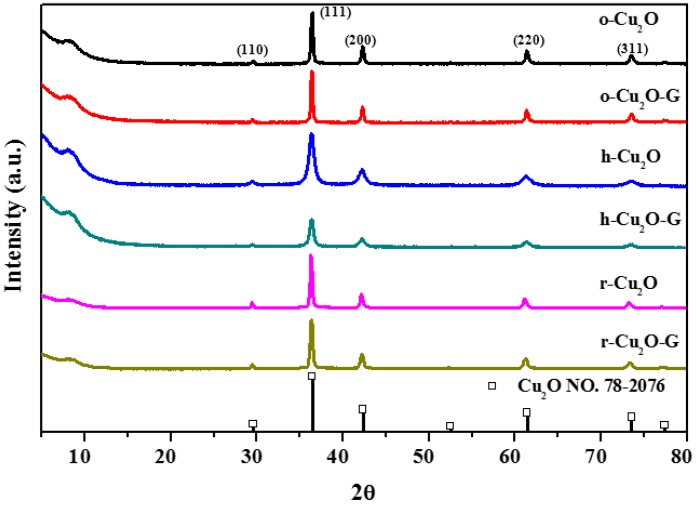
X-ray diffraction (XRD) patterns of different photocatalysts.

As displayed in Figure 1, the XRD patterns of various shapes of Cu_2_O- and Cu_2_O-incorporated graphene samples possess five characteristic reflections at 2θ = 29.6°, 36.4°, 42.3°, 61.4° and 73.6° which are attributed to the (110), (111), (200), (220), (311) planes of cuprous oxide. This result shows the synthesized samples are classified to the cubic phase Cu_2_O (JCPDS No. 78-2076). The sharp diffraction peaks indicate that the high crystallinity of Cu_2_O with different morphologies in all samples can be prepared by using wet-chemical methods. In the previous study [27], the intensity of the (220) peak to that of the (200) peak (*I*_(220)_/*I*_(200)_) is able to be used to evaluate the degree of crystal structure. For instance, the index of *I*_(220)_/*I*_(200)_ is nearly 0.79 in terms of rhombic dodecahedra Cu_2_O. As can be seen in Figure 1, while incorporating graphene onto various crystals of Cu_2_O (o-Cu_2_O-G, h-Cu_2_O-G and r-Cu_2_O-G), the value of *I*_(220)_/*I*_(200)_ is practically unchanged, indicating that no apparent perturbation of crystal structure is observed. This result implies that the introduction of graphene may cause little impact on the growth of nanocrystal Cu_2_O in the samples. 

**Figure 2:**
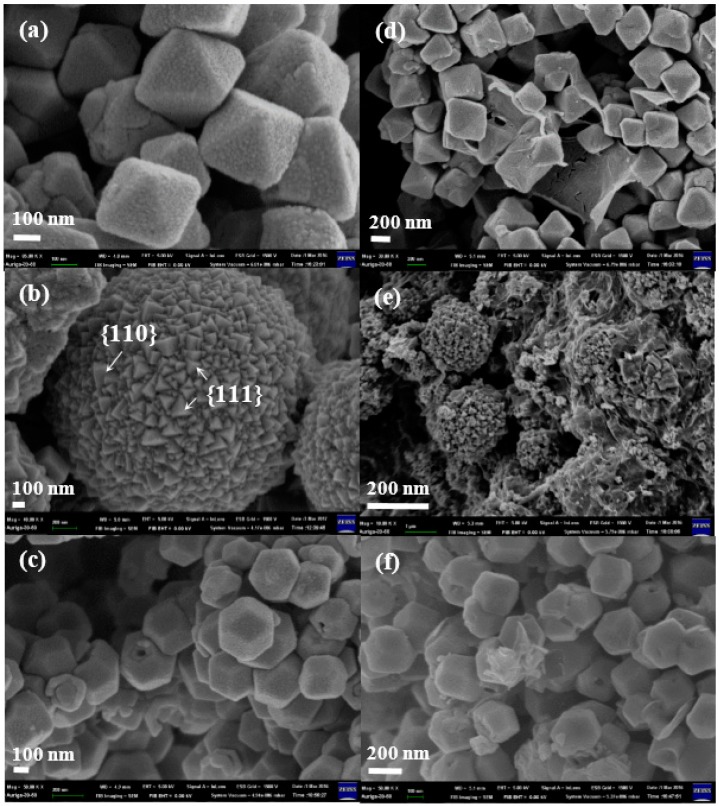
Scanning electron microscope (SEM) images of (**a**) o-Cu_2_O; (**b**) h-Cu_2_O; (**c**) r-Cu_2_O; (**d**) o-Cu_2_O-G; (**e**) h-Cu_2_O-G and (**f**) r-Cu_2_O-G.

**Figure 3:**
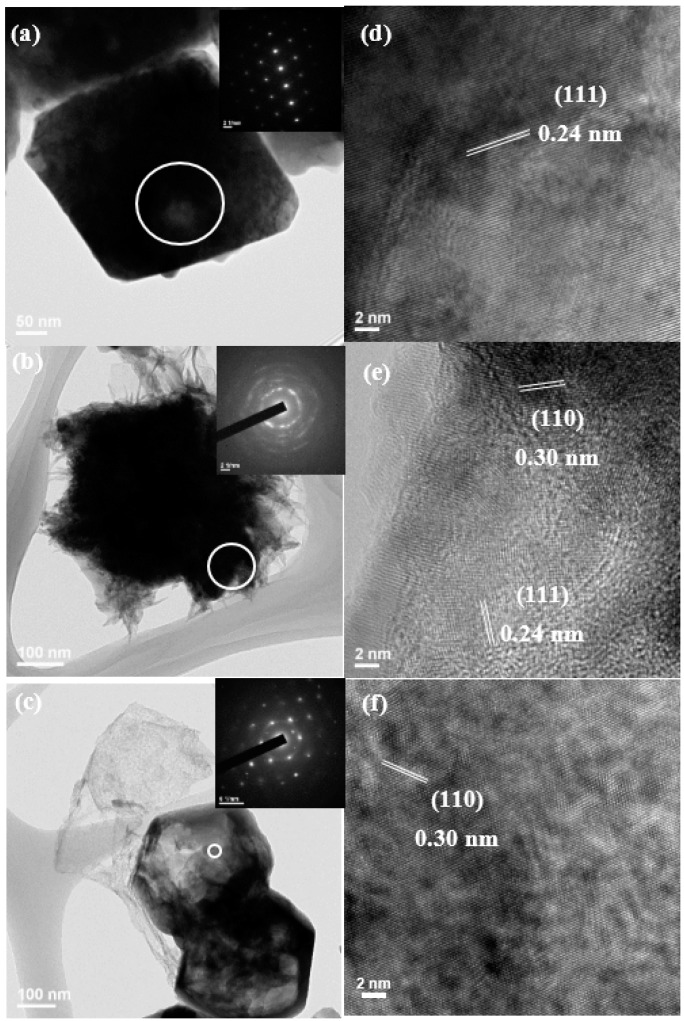
Transmission electron microscope (TEM) images of (**a**) o-Cu_2_O-G; (**b**) h-Cu_2_O-G; (**c**) r-Cu_2_O-G and high-resolution TEM (HRTEM) of (**d**) o-Cu_2_O-G; (**e**) h-Cu_2_O-G; (**f**) r-Cu_2_O-G. Inset: selected area electron diffraction (SAED) from the circle area.

The morphologies of the crystal Cu_2_O, o-Cu_2_O-G, h-Cu_2_O-G and r-Cu_2_O-G were investigated by using field emission scanning electron microscopy (FESEM). As can be seen in Figure 2a, the pristine o-Cu_2_O possesses octahedron morphology of eight {111} facets. Figure 2b presents the typical image of the microspherical h-Cu_2_O with randomly crosslinked polyhedrons which are composed of {110} and {111} facets. In terms of r-Cu_2_O, a 12-facet polyhedral with mostly {110} facets can be observed, as displayed in Figure 2c. Based on the center of the Gaussian distribution, the particle sizes of o-Cu_2_O-G, h-Cu_2_O-G and r-Cu_2_O-G are calculated to be 315 ± 35, 218 ± 19 and 289 ± 38 nm, respectively. Upon incorporating graphene onto various crystals of Cu_2_O (o-Cu_2_O-G, h-Cu_2_O-G and r-Cu_2_O-G), the morphologies and crystal structure Cu_2_O are almost the same as those without graphene, as shown in Figure 2c–e. The microstructures of the Cu_2_O nanocrystals were additionally identified by TEM. It can be observed that the TEM images (Figure 3) show that crystal Cu_2_O is incorporating with the wrinkled, thin and transparent graphene nanosheets. The high-resolution TEM (HRTEM) images of o-Cu_2_O-G, h-Cu_2_O-G and r-Cu_2_O-G in Figure 3d–f show that the interplanar lattice with d-spacings of 0.24 and 0.30 nm are, respectively, assigned to the (111) and (110) planes of Cu_2_O. Moreover, selected area electron diffraction (SAED) and HRTEM suggest that these crystal Cu_2_O samples possess a single-crystal structure, which matches well with the high crystallinity explored by XRD. Therefore, different shapes of highly crystallized Cu_2_O decorated with graphene nanosheets have been fabricated by using simple wet-chemical methods.

**Figure 4:**
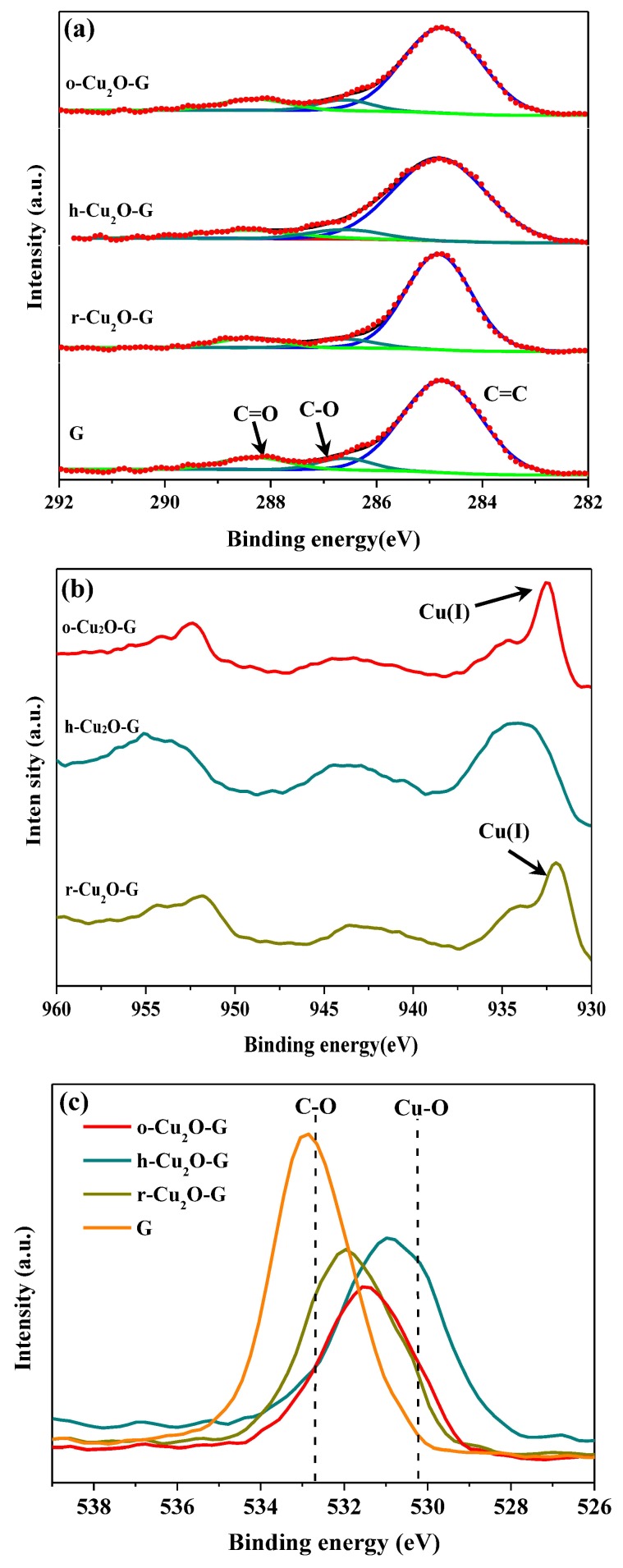
X-ray photoelectron spectroscopy (XPS) spectra of different photocatalysts at the (**a**) high-resolution C 1s core-level; (**b**) Cu 2p core-level; and (**c**) O 1s core-level.

The surface atomic compositions and interfacial electronic states of the samples can be investigated by XPS analysis, as displayed in Figure 4. The peaks at 284.6, 286.5 and 288.4 eV in the C1s spectra (Figure 4a) are assigned to non-oxygenated, epoxy/hydroxyl and carboxyl carbons, respectively [45,46]. The intensities observed for the peaks at 286.5 and 288.4 eV in the o-Cu_2_O-G, h-Cu_2_O-G and r-Cu_2_O-G samples are decreased slightly in comparison to the pure graphene oxide sheets, suggesting that the graphene sheets may maintain the reduced states even with the existence of Cu_2_O nanocrystals in the samples. The XPS of spectra of Cu2p (Figure 4b) indicate the photocatalysts have the principal and satellite features at ca. 934 eV and 944 eV which are attributed to Cu(II), while the features at ca. 932 and 952 eV are assigned to Cu(I) 2p_3/2_ and Cu(I) 2p_1/2_ peaks [9], respectively. In addition, the O1s (see Figure 4c) peaks at ca. 532.5 and 530.3 eV are assigned to C-O and Cu-O bindings, respectively. As a result, the contents of copper(I) for o-Cu_2_O-G and r-Cu_2_O-G composites are much higher, indicating that Cu_2_O nanoparticles may exist stably when dispersed on graphene nanosheets, which can maintain the photocatalytic performance during photodegradation process [47]. Moreover, it is hard to attain bulk information of the atoms by using XPS because a small number of atomic layers on the surface are identified. In addition, it is noteworthy that the diffraction features of copper oxide could hardly be found for all photocatalysts by using the XRD, which is possibly because of the identification of the crystal structure in the bulk phase (see Figure 1).

**Figure 5:**
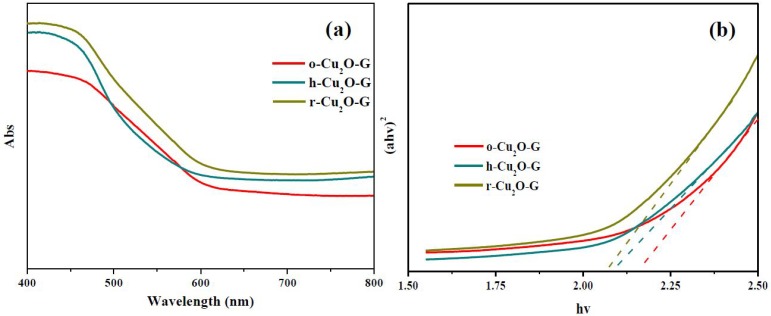
(**a**) Ultraviolet-visible (UV-Vis) absorption spectra for different samples and (**b**) the corresponding Kubelka–Munk plots of photocatalysts.

To understand the optical properties of o-Cu_2_O-G, h-Cu_2_O-G and r-Cu_2_O-G nanocomposites, diffused reflectance UV-Vis (DR UV-Vis) absorption spectroscopies were carried out. Figure 5a shows that o-Cu_2_O-G possesses a band edge of ca. 580 nm in the visible region, suggesting that o-Cu_2_O-G photocatalysts having the bandgap of ca. 2.2 eV (see Figure 5b) should be a visible light-sensitive semiconductor. However, the r-Cu_2_O-G photocatalysts show slightly increased absorbance intensities in the visible-light region (400–800 nm) and absorption edge positions as compared to o-Cu_2_O-G and h-Cu_2_O-G. Moreover, the band gap of these photocatalysts can be obtained by plotting transformed Kubelka–Munk functions, as illustrated in Figure 5b. The band gaps of o-Cu_2_O-G, h-Cu_2_O-G and r-Cu_2_O-G photocatalysts are ca. 2.20, 2.12 and 2.06 eV, respectively. This finding may be due to their different crystal sizes and distinct exposed facets in the photocatalysts. Combining the outcomes of the XRD, TEM, SEM, XPS and DR UV-Vis spectra, various morphologies of crystal Cu_2_O incorporated with graphene nanosheets may be able to take advantage of natural light for decontamination and also be recycled for reuse after photocatalytic reactions.

**Figure 6:**
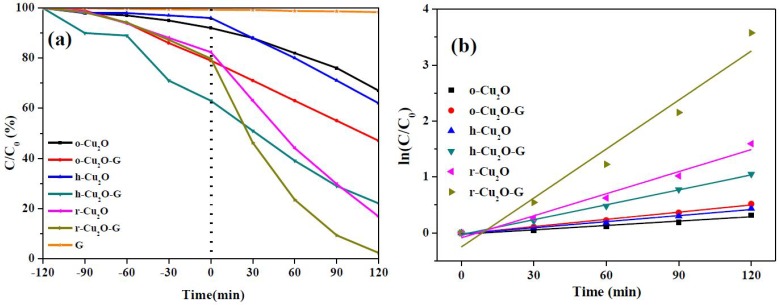
(**a**) Relative concentration of methyl orange (MO) versus time by various photocatalysts under visible light and (**b**) kinetic plots from the data in (**a**).

The photocatalytic performance of pure cuprous oxides (o-Cu_2_O, h-Cu_2_O and r-Cu_2_O) and various graphene-incorporated cuprous oxides (o-Cu_2_O-G, h-Cu_2_O-G and r-Cu_2_O-G) were studied by the photocatalytic oxidation of the MO solution under visible light illumination at room temperature. About 8.3, 4.5, 17.7, 21.2, 37.4 and 20.2% of MO were adsorbed by o-Cu_2_O, h-Cu_2_O, r-Cu_2_O, o-Cu_2_O-G, h-Cu_2_O-G and r-Cu_2_O-G after 120 min in the dark, respectively, indicating that the presence of a high surface area of graphene can increase MO adsorption. Figure 6a shows the concentration variations of MO as a function of irradiation time. It can be seen that little photocatalytic degradation of MO on the graphene is apparent. The order of MO photodegradation ratios of r-Cu_2_O (79.4%) > h-Cu_2_O (35.4%) > o-Cu_2_O (26.9%) can be observed after 120 min of visible light irradiation. Based on this result, it is concluded that the rhombic dodecahedra Cu_2_O nanocrystals which expose mainly {110} facets exhibit superior photocatalytic activity. However, the octahedral Cu_2_O composed of {111} facets have the lowest photocatalytic activity. The hierarchical facets of Cu_2_O exposing both {110} and {111} facets possess moderate photodegradation activity. Upon the incorporation of graphene sheets onto the aforementioned Cu_2_O crystals, all the photoactivities of prepared catalysts are highly enhanced, suggesting that the existence of graphene owing to the unique interface contact between Cu_2_O facets and graphene [48] can enhance the photocatalytic efficiency. Among them, ca. 98% photodegradation of MO can be observed for the r-Cu_2_O-G photocatalysts. It has been reported that the level of conduction band (CB) potential for Cu_2_O (−3.0 eV vs. vacuum) [48] was greater than the level of lowest unoccupied molecular orbital (LUMO) for MO (−3.3 eV vs. vacuum) [49]. As a result, the electron transfer from the excited MO to the Cu_2_O can barely happen, i.e., the MO sensitization during photocatalysis should hardly occur. Moreover, kinetic studies can be used to confirm the photocatalytic activities of various catalysts. As can be seen in Figure 6b, the photocatalytic reactions follow pseudo-first order kinetics and the corresponding data for rate constants (k) can be attained. Consequently, the k values of o-Cu_2_O, h-Cu_2_O, r-Cu_2_O, o-Cu_2_O-G, h-Cu_2_O-G and r-Cu_2_O-G are found to be ca. 0.0026, 0.0036, 0.0131, 0.0043, 0.0089 and 0.0292 min^−1^, respectively. It should be noted that the photodegradation rate of r-Cu_2_O-G is 6.8 and 3.3 times higher than those of o-Cu_2_O-G and h-Cu_2_O-G, respectively.

**Figure 7:**
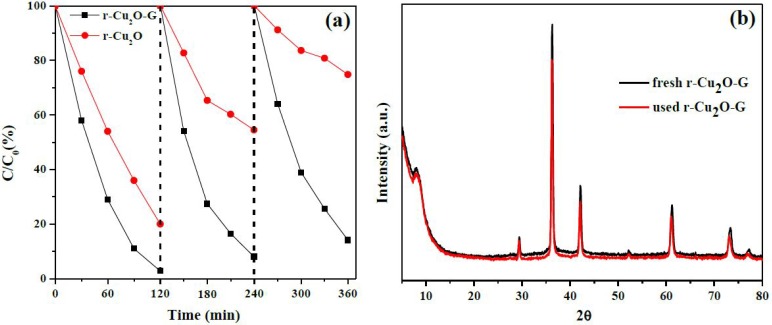
(**a**) Cyclic tests of r-Cu_2_O and r-Cu_2_O-G photocatalysts for MO photodegradation under visible light; (**b**) XRD patterns of the fresh and used r-Cu_2_O-G.

It is crucial to explore the durability and reusability in terms of practical applications of these photocatalysts. We take r-Cu_2_O and r-Cu_2_O-G as the example photocatalysts to test the stability of MO degradation. As can be seen in Figure 7a, an obvious decrease of photocatalytic performance (ca. 54.8%, i.e., from 80.0 to 25.2%) can be found for r-Cu_2_O after three consecutive tests. However, only ca. 10% of efficiency decline has been observed for r-Cu_2_O-G, which is probably due to the assistance of graphene. It is noteworthy that no obvious dissimilarity can be found in the fresh and used r-Cu_2_O-G photocatalysts (see XRD patterns in Figure 7b) in which Cu_2_O is still the dominant species. The slight decline of catalytic performance in the r-Cu_2_O-G photocatalysts may be due to the occurrence of intermediates [46] during photoreaction. It should be noted that the synthesized r-Cu_2_O-G photocatalysts can perform the photodegradation of MO with the highest kinetic rate (degradation efficiency = ca. 98% within 120 min) upon the presence of ultra-low content of samples (0.06 g L^−1^) using visible light illumination. Moreover, these r-Cu_2_O-G photocatalysts can be obtained via a facile and low-cost liquid-phase method. Above all, our r-Cu_2_O-G nanocomposites also have a remarkable enhancement of their long-term durability by the assistance of 1 wt % of graphene. In comparison to previously reported Cu_2_O-based photocatalysts, the synthesized r-Cu_2_O-G photocatalysts with dominant {110} facets of crystal Cu_2_O exhibit an excellent degradation kinetic in a low concentration of photocatalysts under visible-light irradiation that could be practically used to make the best use of daylight for the remediation of organic wastewater.

**Figure 8:**
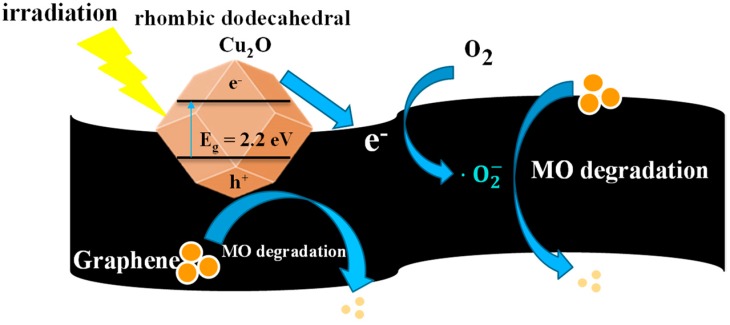
Possible mechanism of MO photodegradation over r-Cu_2_O-G under visible-light illumination.

As shown in Figure 8, according to the aforementioned results a probable mechanism for MO degradation by crystal Cu_2_O-graphene photocatalysts can be proposed. Upon visible-light illumination, the crystal Cu_2_O (e.g., rhombic dodecahedra Cu_2_O) is excited to produce electrons (in the conduction band (CB)) and holes (in the valence band (VB)). Because of the stronger contact between crystal facets and the graphene surface, the photogenerated electrons can be rapidly transported to the graphene nanosheets. As a consequence of the small difference between the Fermi potential of graphene and the reduction potential of O_2_/O_2_^−^ [50], O_2_^−^ and H_2_O_2_ can be observed after the reaction of high-energy electrons with the dissolved oxygen. In this way, the recombination of electron/hole pairs can be greatly prohibited. In addition, compared to octahedral Cu_2_O (mainly {111} facets), the holes (h^+^) with positive charge generated on the valence band (ca. 1.92 eV vs. normal hydrogen electrode (NHE)) of rhombic dodecahedra Cu_2_O (mainly {110} facets) also can degrade more MO molecules (ca. 1.48 eV vs. NHE) [51,52] since the number of dangling Cu atoms on the {110} plane per unit surface area was nearly 1.5 times higher than that on the {111} plane, resulting in a more positively charged surface on {110} facets [28]. Therefore, the superoxide radical anions, hydrogen peroxide and h^+^ may be the dominant species governing the photodegradation of MO under visible-light irradiation. It should be noted that the graphene sheets not only serve as acceptors of the photogenerated electrons from Cu_2_O but also as stabilizers to prevent the crystal Cu_2_O from structural destruction under irradiation over a long period.

## 4. Conclusions

In this study, three different crystal facets of Cu_2_O photocatalysts decorated with graphene sheets were synthesized by using simple solution-phase methods. The order in terms of photocatalytic MO degradation was observed to be as follows: rhombic dodecahedral Cu_2_O-graphene (r-Cu_2_O-G) > hierarchical Cu_2_O-graphene (h-Cu_2_O-G) > octahedral Cu_2_O-graphene (o-Cu_2_O-G), which was demonstrated to be related to the morphologies and crystal structure of facets (i.e., the {110} facets are most active toward MO degradation). In earlier reports regarding Cu_2_O-based photocatalysts, the r-Cu_2_O-G photocatalysts possessed high kinetics of photocatalytic degradation, i.e., by using an ultra-low content of samples (0.06 g L^−1^) to reach 98% of MO photodegradation under 120 min irradiation of visible light. More importantly, the cycling tests indicate that the resulting r-Cu_2_O-G composites show a surpassing durability compared to pure r-Cu_2_O nanocrystals. These significant enhancements are possibly because of the unique interfacial interaction of rhombic dodecahedra Cu_2_O (more positively charged {110} facets) with the graphene nanosheets, which could lead to the effective isolation of electron/hole pairs, stabilization of the crystal Cu_2_O, and an increase of MO adsorption. Consequently, the development of a cost-effective and facile method to prepare r-Cu_2_O-G composites with mostly {110} facets and graphene sheets, which exhibit superior photocatalytic performance (kinetics and stability), offers the potential for a promising application in the treatment of organic wastewater by utilizing natural sunlight.

## References

[1] Gilja V., Novakovic K., Travas-Sejdic J., Hrnjak-Murgic Z., Rokovic M.K., Zic M. (2018). Stability and synergistic effect of polyaniline/TiO_2_ photocatalysts in degradation of Azo Dye in wastewater. Nanomaterials.

[2] Liao T.W., Verbruggen S.W., Claes N., Yadav A., Grandjean D., Bals S., Lievens P. (2018). TiO_2_ films modified with Au nanoclusters as self-cleaning surfaces under visible light. Nanomaterials.

[3] Ye L.Q., Su Y.R., Jin X.L., Xie H.Q., Zhang C. (2014). Recent advances in BiOX (X = Cl, Br and I) photocatalysts: Synthesis, modification, facet effects and mechanisms. Environ. Sci. Nano.

[4] Liu S.-H., Syu H.-R. (2012). One-step fabrication of N-doped mesoporous TiO_2_ nanoparticles by self-assembly for photocatalytic water splitting under visible light. Appl. Energy.

[5] Pang D.D., Wang Y.T., Ma X.D., Ouyang F. (2014). Fluorine promoted and silica supported TiO_2_ for photocatalytic decomposition of acrylonitrile under simulant solar light irradiation. Chem. Eng. J..

[6] Liu S.-H., Syu H.-R. (2013). High visible-light photocatalytic hydrogen evolution of C,N-codoped mesoporous TiO_2_ nanoparticles prepared via an ionic-liquid template approach. Int. J. Hydrogen Energy.

[7] Song X.L., Li Y.Y., Wei Z.D., Ye S.Y., Dionysiou D.D. (2017). Synthesis of BiVO_4_/P25 composites for the photocatalytic degradation of ethylene under visible light. Chem. Eng. J..

[8] Liu Y.Z., Xu J.A., Wang L.Q., Zhang H.Y., Xu P., Duan X.G., Sun H.Q., Wang S.B. (2017). Three-dDimensional BiOI/BiOX (X = Cl or Br) nanohybrids for enhanced visible-light photocatalytic activity. Nanomaterials.

[9] Tian L.Y., Rui Y.L., Sun K.L., Cui W.Q., An W.J. (2018). Surface decoration of ZnWO_4_ nanorods with Cu_2_O nanoparticles to bBuild heterostructure with enhanced photocatalysis. Nanomaterials.

[10] Kumar A., Kumar A., Sharma G., Al-Muhtaseb A.H., Naushad M., Ghfar A.A., Stadler F.J. (2018). Quaternary magnetic BiOCl/g-C_3_N_4_/Cu_2_O/Fe_3_O_4_ nano-junction for visible light and solar powered degradation of sulfamethoxazole from aqueous environment. Chem. Eng. J..

[11] Singh M., Jampaiah D., Kandjani A.E., Sabri Y.M., Della Gaspera E., Reineck P., Judd M., Langley J., Cox N., van Embden J. (2018). Oxygen-deficient photostable Cu_2_O for enhanced visible light photocatalytic activity. Nanoscale.

[12] Sakar M., Balakumar S. (2018). Reverse Ostwald ripening process induced dispersion of Cu_2_O nanoparticles in silver-matrix and their interfacial mechanism mediated sunlight driven photocatalytic properties. J. Photochem. Photobiol. A.

[13] Wei Q., Wang Y., Qin H.Y., Wu J.M., Lu Y.F., Chi H.Z., Yang F., Zhou B., Yu H.L., Liu J.B. (2018). Construction of rGO wrapping octahedral Ag-Cu_2_O heterostructure for enhanced visible light photocatalytic activity. Appl. Catal. B Environ..

[14] Su Y., Li H.F., Ma H.B., Wang H., Robertson J., Nathan A. (2018). Dye-assisted transformation of Cu_2_O nanocrystals to amorphous Cu_x_O nanoflakes for enhanced photocatalytic performance. ACS Omega.

[15] Sun S.D. (2015). Recent advances in hybrid Cu_2_O-based heterogeneous nanostructures. Nanoscale.

[16] Chen X.Q., Wu Z.S., Gao Z.Z., Ye B.C. (2018). Effect of different activated carbon as carrier on the photocatalytic activity of Ag-N-ZnO photocatalyst for methyl orange degradation under visible light irradiation. Nanomaterials.

[17] Choi H., Shin D., Yeo B.C., Song T., Han S.S., Park N., Kim S. (2016). Simultaneously controllable doping sites and the activity of a W-N codoped TiO_2_ photocatalyst. ACS Catal..

[18] Luster E., Avisar D., Horovitz I., Lozzi L., Baker M.A., Grilli R., Mamane H. (2017). N-doped TiO_2_-coated ceramic membrane for carbamazepine degradation in different water qualities. Nanomaterials.

[19] Bailón-García E., Elmouwahidi A., Álvarez M.A., Carrasco-Marín F., Pérez-Cadenas A.F., Maldonado-Hóda F.J. (2017). New carbon xerogel-TiO_2_ composites with high performance as visible-light photocatalysts for dye mineralization. Appl. Catal. B Environ..

[20] Klaysri R., Ratova M., Praserthdam P., Kelly P.J. (2017). Deposition of visible light-active C-doped titania films via magnetron sputtering using CO_2_ as a source of carbon. Nanomaterials.

[21] Nica I.C., Stan M.S., Dinischiotu A., Popa M., Chifiriuc M.C., Lazar V., Pircalabioru G.G., Bezirtzoglou E., Iordache O.G., Varzaru E. (2016). Innovative self-cleaning and biocompatible polyester textiles nano-decorated with Fe-N-doped titanium dioxide. Nanomaterials.

[22] Reddy P.A.K., Reddy P.V.L., Kwon E., Kim K.H., Akter T., Kalagara S. (2016). Recent advances in photocatalytic treatment of pollutants in aqueous media. Environ. Int..

[23] Chang M.L., Hu H.W., Zhang Y.Y., Chen D.C., Wu L.P., Li X.J. (2017). Improving visible light-absorptivity and photoelectric conversion efficiency of a TiO_2_ nanotube anode film by sensitization with Bi_2_O_3_ Nanoparticles. Nanomaterials.

[24] Singh R., Dutta S. (2018). A review on H_2_ production through photocatalytic reactions using TiO_2_/TiO_2_-assisted catalysts. Fuel.

[25] Hu J.L., Tu J.H., Li X.Y., Wang Z.Y., Li Y., Li Q.S., Wang F.P. (2017). Enhanced UV-Visible light photocatalytic activity by constructing appropriate heterostructures between mesopore TiO_2_ nanospheres and Sn_3_O_4_ nanoparticles. Nanomaterials.

[26] Petronella F., Truppi A., Ingrosso C., Placido T., Striccoli M., Curri M.L., Agostiano A., Comparelli R. (2017). Nanocomposite materials for photocatalytic degradation of pollutants. Catal. Today.

[27] Huang W.-C., Lyu L.-M., Yang Y.-C., Huang M.H. (2012). Synthesis of Cu_2_O nanocrystals from cubic to rhombic dodecahedral structures and their comparative photocatalytic activity. J. Am. Chem. Soc..

[28] Shang Y., Guo L. (2015). Facet-controlled synthetic strategy of Cu_2_O-based crystals for catalysis and sensing. Adv. Sci..

[29] Yuan G.-Z., Hsia C.-F., Lin Z.-W., Chiang C., Chiang Y.-W., Huang M.H. (2016). Highly facet-dependent photocatalytic properties of Cu_2_O crystals established through the formation of Au-decorated Cu_2_O heterostructures. Chem. Eur. J..

[30] Chen D.S., Yu W.B., Deng Z., Liu J., Jin J., Li Y., Wu M., Chen L.H., Su B.L. (2015). Hollow Cu_2_O microspheres with two active {111} and {110} facets for highly selective adsorption and photodegradation of anionic dye. RSC Adv..

[31] Zhang Y., Deng B., Zhang T.R., Gao D.M., Xu A.W. (2010). Shape effects of Cu_2_O polyhedral microcrystals on photocatalytic activity. J. Phys. Chem. C.

[32] Xu L., Zhang F.Y., Song X.Y., Yin Z.L., Bu Y.X. (2015). Construction of reduced graphene oxide-supported Ag-Cu_2_O composites with hierarchical structures for enhanced photocatalytic activities and recyclability. J. Mater. Chem. A.

[33] Babu S.G., Vinoth R., Narayana P.S., Bahnemann D., Neppolian B.S. (2015). Reduced graphene oxide wrapped Cu_2_O supported on C_3_N_4_: An efficient visible light responsive semiconductor photocatalyst. APL Mater..

[34] Yu L., Li G.J., Zhang X.S., Ba X., Shi G.D., Li Y., Wong P.K., Yu J.C., Yu Y. (2016). Enhanced activity and stability of carbon-decorated cuprous oxide mesoporous nanorods for CO_2_ reduction in artificial photosynthesis. ACS Catal..

[35] Liu S.-H., Wei Y.-S., Lu J.-S. (2016). Visible-light-driven photodegradation of sulfamethoxazole and methylene blue by Cu_2_O/rGO photocatalysts. Chemosphere.

[36] Upadhyay R.K., Soin N., Roy S.S. (2014). Role of graphene/metal oxide composites as photocatalysts, adsorbents and disinfectants in water treatment: A review. RSC Adv..

[37] Stoller M.D., Park S., Zhu Y., An J., Ruoff R.S. (2008). Graphene-based ultracapacitors. Nano Lett..

[38] Mayorov A.S., Gorbachev R.V., Morozov S.V., Britnell L., Jalil R., Ponomarenko L.A., Blake P., Novoselov K.S., Watanabe K., Taniguchi T. (2011). Micrometer-scale ballistic transport in encapsulated graphene at room temperature. Nano Lett..

[39] Chen Y., Sun H.Q., Peng W.C. (2017). 2D transition metal dichalcogenides and graphene-based ternary composites for photocatalytic hydrogen evolution and pollutants degradation. Nanomaterials.

[40] Tian Y., Sun Z.H., Zhang Y.G., Wang X., Bakenov Z., Yin F.X. (2018). Micro-spherical sulfur/graphene oxide composite via spray drying for high performance lithium sulfur batteries. Nanomaterials.

[41] Gong Y.X., Wang Y., Sun G., Jia T.K., Jia L., Zhang F.M., Lin L., Zhang B.Q., Cao J.L., Zhang Z.Y. (2018). Carbon nitride decorated ball-flower like Co_3_O_4_ hybrid composite: Hydrothermal synthesis and ethanol gas sensing application. Nanomaterials.

[42] Zhou Y.X., Jia L.P., Wang T.X., Du Y.L., Wang C.M. (2018). Preparation of carbon nanotube and graphene doped polyphenylene sulfide flexible film electrodes and the electrodeposition of Cu_2_O nanocrystals for hydrogen-generation. Int. J. Hydrogen Energy.

[43] Sharma K., Maiti K., Kim N.H., Hui D., Lee J.H. (2018). Green synthesis of glucose-reduced graphene oxide supported Ag-Cu_2_O nanocomposites for the enhanced visible-light photocatalytic activity. Compos. Part B.

[44] González J.A., Villanueva M.E., Piehl L.L., Copello G.J. (2015). Development of a chitin/graphene oxide hybrid composite for the removal of pollutant dyes: Adsorption and desorption study. Chem. Eng. J..

[45] Stankovich S., Dikin D.A., Piner R.D., Kohlhaas K.A., Kleinhammes A., Jia Y., Wu Y., Nguyen S.T., Ruoff R.S. (2007). Synthesis of graphene-based nanosheets via chemical reduction of exfoliated graphite oxide. Carbon.

[46] Liu S.-H., Yang S.-W. (2018). Highly efficient cuprous oxide nanocrystals assisted with graphene for decolorization using visible light. Water Air Soil Pollut..

[47] Pu Y.-C., Chou H.-Y., Kuo W.-S., Wei K.-H., Hsu Y.-J. (2017). Interfacial charge carrier dynamics of cuprous oxide-reduced graphene oxide (Cu_2_O-rGO) nanoheterostructures and their related visible-light-driven photocatalysis. Appl. Catal. B Environ..

[48] Gao Z., Liu J., Xu F., Wu D., Wu Z., Jiang K. (2012). One-pot synthesis of graphene-cuprous oxide composite with enhanced photocatalytic activity. Solid State Sci..

[49] Chang X.F., Gondal M.A., Al-Saadi A.A., Ali M.A., Shen H., Zhou Q., Zhang J., Du M., Liu Y., Ji G. (2012). Photodegradation of Rhodamine B over unexcited semiconductor compounds of BiOCl and BiOBr. J. Colloid Interface Sci..

[50] Wang J.T.W., Ball J.M., Barea E.M., Abate A., Alexander-Webber J.A., Huang J., Saliba M., Mora-Sero I., Bisquert J., Snaith H.J. (2014). Low-temperature processed electron collection layers of graphene/TiO_2_ nanocomposites in thin film perovskite solar cells. Nano Lett..

[51] Yan S.C., Li Z.S., Zou Z.G. (2010). Photodegradation of Rhodamine B and methyl orange over boron-doped g-C_3_N_4_ under visible light irradiation. Langmuir.

[52] Hu X., Zhou X., Wang R., Hu C., Qu J. (2014). Characterization and photostability of Cu_2_O-Ag-AgBr/Al_2_O_3_ for the degradation of toxic pollutants with visible-light irradiation. Appl. Catal. B.

